# Secure multiparty quantum computation based on Lagrange unitary operator

**DOI:** 10.1038/s41598-020-64538-8

**Published:** 2020-05-13

**Authors:** Xiuli Song, Rui Gou, Aijun Wen

**Affiliations:** 1 Chongqing University of Posts and Telecommunications, School of Cyber Security and Information Law, Chongqing, 400065 China; 2 Chongqing University of Posts and Telecommunications, School of Computer Science and Technology, Chongqing, 400065 China

**Keywords:** Quantum information, Quantum simulation

## Abstract

As an important subtopic of classical cryptography, secure multiparty quantum computation allows multiple parties to jointly compute their private inputs without revealing them. Most existing secure multiparty computation protocols have the shortcomings of low computational efficiency and high resource consumption. To remedy these shortcomings, we propose a secure multiparty quantum computation protocol by using the Lagrange unitary operator and the Shamir (*t*, *n*) threshold secret sharing, in which the server generates all secret shares and distributes each secret share to the corresponding participant, in addition, he prepares a particle and sends it to the first participant. The first participant performs the Lagrange unitary operation on the received particle, and then sends the transformed particle to the next participant. Until the last participant’s computation task is completed, the transformed particle is sent back to the server. The server performs Lagrange unitary operation on the received particle by using a secret message, and then measures the transformed particle to obtain the sum of the calculations of multiple participants. Security analysis shows that the proposed protocol can resist intercept-measurement attack, intercept-resend attack, entanglement-swapping attack, entanglement-measurement attack and collusion attack. Performance comparison shows that it has higher computation efficiency and lower resource consumption than other similar protocols.

## Introduction

As an important part of classical cryptography, classical secure multiparty computation (CSMC) allows two or more participants cooperate to calculate a relevant function without disclosing their private input information to each other, and finally output a calculation result. The CSMC comes from the millionaire problem of Yao^1^. Based on this problem, many CSMC protocols were proposed^2^. Nowadays, CSMC is widely applied to electronic transactions, information retrieval, data mining and other fields. With the rapid development of quantum communication and quantum computation, the security of classical cryptography has been greatly challenged, and CSMC is no exception. Quantum secure multi-party computation (QSMC)^3–17^ is the expansion of CSMC to the quantum field. It overcomes the security defects of CSMC in theft detection and has the advantages beyond the reach of CSMC.

To date, many researchers have investigated QSMC. In 2002, Crepeau *et al*.^18^ proposed a multiparty quantum computation which can get right results as long as the number of dishonest players is less than *n*/6. In 2006, Ben-Or *et al*.^19^ studied how many participants must remain honest in order for the right results in QSMC. In 2008, Ivan *et al*.^20^ proposed the first general protocol for QSMC, in which the total workload required by $n$ players to compute a function $f$ only relates with the growth of $n$. In 2010, Dominique^21^ proposed quantum universal composability model (UC) secure protocol for general multiparty computation can be constructed from commitment. In 2012, Li *et al*.^22^ proposed a secure two-party scalar product protocol which takes advantage of quantum entanglement, quantum measurement and trusted third party (TP). In 2013, Li *et al*.^23^ proposed a QSMC protocol via quantum entanglement states. In 2019, Shi^24^ proposed a generic quantum protocol for one-sided secure two-party classical computations, in which two parties can privately compute any classical function theoretically without the help of any third party.

In the process of investigate secure multiparty computation, secure multiparty quantum summation is also being investigated as a branch of secure multiparty quantum computation. In 2002, Heinrich *et al*.^25^ studied the summation of sequences in the quantum computation model. In 2004, Heinrich *et al*.^26^ continued to study the quantum summation algorithm in quantum multiparty computation. In 2007, Du *et al*.^27^ proposed a protocol of secure quantum multiparty addition modulo $n+1(n\ge 6)$ by using non-orthogonal states. In 2010, Chen *et al*.^28^ proposed a quantum addition protocol based on GHZ states. In 2014, Zhang *et al*.^29^ proposed a quantum summation protocol by the particles in both polarization and spatial-mode degrees of freedom. In 2015, Zhang *et al*.^30^ proposed a quantum summation protocol base on the genuinely maximally entangled six-qubit states.

In recent years, Shi *et al*.^31^ proposed a multiparty quantum summation and multiplication by quantum Fourier transform. We focus on the first protocol of quantum multiparty summation in the paper. The first participant prepares two initial particle and then he performs $QFT$ and $CNOT$ operations on the two initial particle to generate a 2-particle entangled state, further he sends a particle of entangled state to the next participant. After receiving the particle, the participant prepares a new particle embedding the private information, and then he performs the unitary operations on the received particle and the prepared particle. The transformed particle is sent to the next participant. After all participants have completed their computation tasks, the first participant uses $QF{T}^{-1}$ operation to obtain the sum of the privacy data of all participants. In this protocol, each participant needs to prepare initial particles, so it has a high resource consumption problem. Clementi *et al*.^32^ proposed a protocol to perform multiparty computing among parties with limited quantum computation resources. In this protocol, all participants used only classical linear computations and finite quantum resources to jointly compute a nonlinear multivariable function *f*(${x}_{1},{x}_{2},\ldots ,{x}_{n}$). The protocol is on two level Hilbert space, so it has a insufficient universality and practicability problem. Yang *et al*.^33^ propose secure multiparty quantum summation protocol based on quantum Fourier transform. In this protocol, The first participant prepares $n$ entangled states, each of which has $n$ particles. Each participant has $n$ privacy data and receives $n$ quantum sequences from dealer, and then he embeds the $n$ privacy data into the received quantum sequence by performing $QFT$ operation and unitary operation. After all participants have completed their computation tasks, each participant performs a measurement operations on the particle of entangled states to obtain the computation result. The protocol needs to prepare many initial particles and performs many $QFT$ operations and unitary operations, so it has a high resource consumption cost and high computation cost problem.

In order to reduce the resource cost and the computation cost, increase universality and practicality, this paper proposes a $d$-dimensional security multiparty quantum computation protocol based on Lagrange unitary operator, which completes summation of computational result of multiple participants. Shamir’s $(t,n)$ threshold scheme is used to enhance the security of the proposed protocol. Finally, the server obtains the summation result of multiple participants by mesuring the particle. On the one hand, the correctness of the proposed protocol has proved theoretically, on the other hand, simulation experiments further verify the correctness of the proposed protocol.

Compared with other similar protocols, the proposed protocol is on the *d*-dimensional ($d\ge 2$) quantum space. When the quantum environment is free space, it has better universality and practicability than the 2-dimensional QSMC protocol. What’s more, each participant only needs to perform an unitary operation, which means the proposed protocol is higher computation efficiency than other similar protocols. At last, only one quantum measurement is performed, and an initial particle is prepared in the proposed protocol, which means the proposed protocol is lower resource consumption cost than other similar protocols.

The rest of this paper is organized as follows. In preliminaries, we introduce the preliminary knowledge used in this paper. In results, we describe the proposed protocol. In correctness proof, we prove the correctness of the proposed protocol; In simulation, we prove the proposed protocol’s correctness by simulation. In security analysis, we analyze the security of the proposed protocol. In performance analysis and comparison, we analyze and compare the proposed protocol with other similar protocols. Finally, conclusion is given.

## Preliminaries

In this section, the related preliminaries are introduced including Lagrange unitary operator and Shamir’s ($t,n$) threshold scheme, which will be used in presenting proposed protocol.

## Lagrange unitary operator

Suppose that there are $n$ distinct points set {(${x}_{i},{y}_{i}$)|$i=1,2,\ldots ,n$} satisfying ${y}_{i}=f$(*x*_*i*_), which can be constructed as a polynomial with $n-1$ degree

1 $$p(x)=\sum _{j}\frac{{\prod }_{k\ne j}(x-{x}_{k})}{{\prod }_{k\ne j}({x}_{j}-{x}_{k})}{y}_{j}.$$

Suppose that $q$ is a $n\times n$ unitary matrix, any of the set $\{{q}^{1},{q}^{2},{q}^{3},\ldots ,{q}^{n-1}\}$ is not equal to unit matrix $u$, and *q*^0^ is a $n\times n$ unit matrix $u$. The set $\{{q}^{0},{q}^{1},{q}^{2},\ldots ,{q}^{n-1}\}$ constitutes a finite cyclic matrix group. According on Eq. (1), the Lagrange unitary operator^34^ $m(\theta )$ can be defined by

2 $$m(\theta )=\sum _{j}\frac{{\prod }_{k\ne j}({e}^{i\theta }-{\omega }^{k})}{{\prod }_{k\ne j}({\omega }^{j}-{\omega }^{k})}{q}^{j},$$

where $$\omega ={e}^{i2\pi /n}$$, $$j;k\in \{0,1,\ldots ,n-1\}$$.

For example, when $q$ is a 3 × 3 matrix, the set {${q}^{0},{q}^{1},{q}^{2}$} is constructed as follows

$${q}^{1}=(\begin{array}{ccc}0 & 1 & 0\\ 0 & 0 & 1\\ 1 & 0 & 0\end{array}),\,{q}^{2}=(\begin{array}{ccc}0 & 0 & 1\\ 1 & 0 & 0\\ 0 & 1 & 0\end{array}),\,{q}^{0}=(\begin{array}{ccc}1 & 0 & 0\\ 0 & 1 & 0\\ 0 & 0 & 1\end{array}).$$

Let us substitute ${q}^{0},{q}^{1},{q}^{2}$ into Eq. (2), the Lagrange unitary operator $m(\theta )$ can be represented as

$$\begin{array}{rcl}m(\theta ) & = & \frac{1}{3}[({x}^{2}+x+1){q}^{0}+(\omega {x}^{2}+\omega {x}^{2}+1){q}^{1}+(\omega {x}^{2}+\omega x+1){q}^{2}]\\  & = & \frac{1}{3}(\begin{array}{ccc}{x}^{2}+x+1 & \omega {x}^{2}+\omega {x}^{2}+1 & \omega {x}^{2}+\omega x+1\\ \omega {x}^{2}+\omega x+1 & {x}^{2}+x+1 & \omega {x}^{2}+\omega {x}^{2}+1\\ \omega {x}^{2}+\omega {x}^{2}+1 & \omega {x}^{2}+\omega x+1 & {x}^{2}+x+1\end{array}),\end{array}$$

where $$x={e}^{i\theta },\omega ={e}^{i2\pi /3}$$.

## Shamir’s (*t*, *n*) threshold scheme

Suppose that there is a trusted server and $n$ participants $\{{P}_{i}|i=(1,2,\ldots ,n)\}$, Shamir’s ($t,n$) threshold scheme consists of the following two stages.

Step 1. Secret distribution stage. The server first generates randomly a polynomial with degree $t-1$: *f*(*x*) = ${a}_{0}+{a}_{1}x+{a}_{2}{x}^{2}+\cdots +{a}_{t-1}{x}^{t-1}$ *mod* *d*, where $({a}_{0},{a}_{1},{a}_{2},\ldots ,{a}_{t-1})\in {Z}_{d}^{t}$, and ${a}_{0}$ is a secret message. Then he computes $n$ secret share {*f*(*x*_*i*_)$|$i = 1, 2, …, n}, and further he sends each secret share *f*(${x}_{i}$) for ($i=1,2,\ldots ,n$) to the corresponding to participants ${P}_{i}$ via a secure channel.

Step 2. Secret reconstruction stage. There are $n$ distinct and nonzero points {*f*(*x*_*i*_)$|$i = 1, 2, …, n} on the polynomial *f*(*x*) = ${a}_{0}+{a}_{1}x+{a}_{2}{x}^{2}+\cdots +{a}_{t-1}{x}^{t-1}$. If at least $t$ points {(${x}_{r}$, *f*(${x}_{r}$))|$r=1,2,\ldots ,t$} are given, the polynomial *f*(*x*) can be reconstructed by using the Lagrange interpolation formula as follows

3 $$f(x)=\mathop{\sum }\limits_{r=1}^{t}f({x}_{r})\prod _{1\le j\le t,j\ne r}\frac{{x}_{j}}{{x}_{j}-{x}_{r}}\,{\rm{mod}}\,d.$$

If any $t$ out of the $n$ participants, denoted by $P=\{{P}_{1},{P}_{2},\ldots ,{P}_{t}\}$, take out their secret shares {(${x}_{r}$, *f*(${x}_{r}$)|$r=1,2,\ldots ,t$}. Then the $t$ participants can reconstruct the original secret message ${a}_{0}$ based on the above Eq. (3).

## The proposed protocol

### Protocol purpose

Suppose that there are $n$ participants, each participant has two parameters, and they want to realize the summation task, where the private information of each participant is the result of ${\theta }_{u}\ast z$,

4 $$({\theta }_{{u}_{1}}\ast {z}_{1})+({\theta }_{{u}_{2}}\ast {z}_{2})+\cdots +({\theta }_{{u}_{n}}\ast {z}_{n})=\mathop{\sum }\limits_{i=1}^{n}({\theta }_{{u}_{i}}\ast {z}_{i}),$$

where $\{{\theta }_{{u}_{i}}\in \left\{0,\frac{2\pi }{d},\ldots ,\frac{(d-1)2\pi }{d}\right\}|i=1,2,\ldots ,n\}$ are the participant’s parameter one, $\{{z}_{i}\in \{0,1,\ldots ,d-1\}$ $|i=1,2,\ldots ,n\}$ are the participant’s parameter two and the symbol + denotes addition modulo 2*π*.

For the convenience of calculation, Lagrange unitary operator *m*() is used to realize the collaborative summation task

5 $$m({\theta }_{{u}_{n}}\ast {z}_{n})\cdots m({\theta }_{{u}_{2}}\ast {z}_{2})m({\theta }_{{u}_{1}}\ast {z}_{1})|0\rangle =|R\rangle ,$$

where $|0\rangle$ is an initial particle, $|R\rangle$ is a quantum state with summation result.

To ensure the security of the proposed protocol, each participant’s share angle ${\theta }_{{v}_{i}}(i=1,2,\ldots ,n)$ is added to $m({\theta }_{{u}_{i}}\ast {z}_{i})$ by using Shamir’s ($t,n$) threshold scheme:

6 $$m(({\theta }_{{u}_{i}}\ast {z}_{n})+{\theta }_{{v}_{n}})\cdots m(({\theta }_{{u}_{2}}\ast {z}_{2})+{\theta }_{{v}_{2}})m(({\theta }_{{u}_{1}}\ast {z}_{1})+{\theta }_{{v}_{1}})|0\rangle =|{R}_{n}\rangle ,$$

where $|{R}_{n}\rangle$ is a quantum state with the summation result after blindness.

### Protocol description

**Figure 1:**
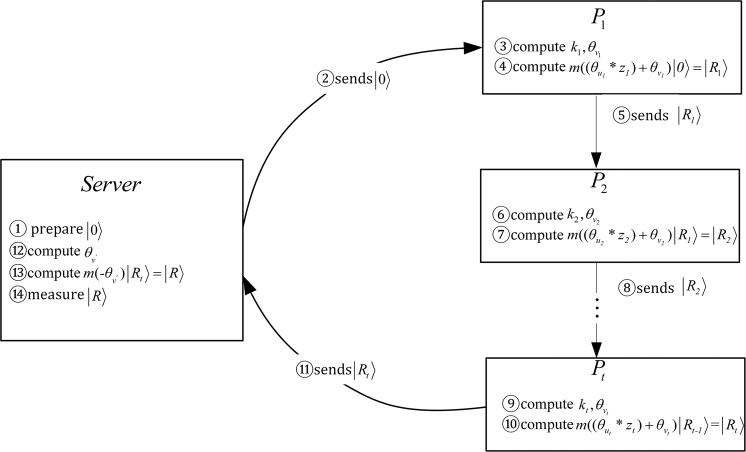
Multiparty computation flow chart.

Based on Shamir’s ($t,n$) threshold scheme, there is a trusted server and $n$ participants $P=\{{P}_{1},{P}_{2},\ldots ,{P}_{n}\}$, any $t$ of $n$ participants want to complete joint computation. The proposed protocol can be divided into three stage: initialization stage, privacy computational stage and result output stage, as is shown in Fig. 1.

### Initialization stage

First, the server computes all secret shares and distribute each secret share to the corresponding participant, in addition prepares an initial particle and sends it to the first participant. This stage consists of the following two steps:

Step 1. The server randomly chooses $t$ privacy integers ${a}_{0},{a}_{1},\ldots ,{a}_{t-1}$($a\in {Z}_{d}^{t}$) and $n$ public distinct and no zero integers ${x}_{1},{x}_{2},\ldots ,{x}_{n}$. Then he constructs a polynomial with degree $t-1$: *f*(*x*) = ${a}_{0}+{a}_{1}x+{a}_{2}{x}^{2}+\cdots +{a}_{t-1}{x}^{t-1}$ *mod* *d* and obtains the set $\{f({x}_{1}),f({x}_{2}),\ldots ,f({x}_{n})\}$. Further, he distributes each secret share *f*(*x*_*i*_)($i=1,2,\ldots ,n$) to the corresponding participant via a secure channel. Here *f*(0) = *a*_0_ is a secret message.

Step 2. The server prepares an initial particle $|0\rangle$ and sends it to the first participant ${P}_{1}$, corresponding to step ①–② in the Fig. 1.

### Privacy computation stage

After the current participant ${P}_{r}$($r\in \{1,2,\ldots ,t\}$) receives the particle sent by the previous participant ${P}_{r-1}$, he performs the Lagrange unitary operation $m(({\theta }_{{u}_{r}}\ast {z}_{r})+{\theta }_{{v}_{r}})$ on the received particle, and then sends the transformed particle to the next participant. This stage consists of the following two steps:

Step 1. ${P}_{1}$ first computes the secret shadow ${k}_{1}=f({x}_{1}){\prod }_{1\le m\le t,m\ne 1}\frac{{x}_{m}}{{x}_{m}-{x}_{1}}\,mod\,d$ and ${\theta }_{{v}_{1}}=\frac{2\pi {k}_{1}}{d}\,mod\,2\pi$. Further, ${P}_{1}$ performs Lagrange unitary operation $m(({\theta }_{{u}_{1}}\,\ast \,{z}_{1})+{\theta }_{{v}_{1}})$ on $|0\rangle$ to obtain the result $|{R}_{1}\rangle$ and sends it to the participant ${P}_{2}$, corresponding to step ③–⑤ in the Fig. 1.

Step 2. Similar to ${P}_{1}$, each of the remaining participants ${P}_{r}$($r\in \{2,3,\ldots ,t\}$) first computes the secret shadow ${k}_{r}=f({x}_{r}){\prod }_{1\le m\le t,m\ne i}\frac{{x}_{m}}{{x}_{m}-{x}_{r}}\,mod\,d$ and ${\theta }_{{v}_{r}}=\frac{2\pi {k}_{r}}{d}mod\,2\pi$. Further, ${P}_{r}$ performs Lagrange unitary operation $m(({\theta }_{{u}_{r}}\,\ast \,{z}_{r})+{\theta }_{{v}_{r}})$ on $|{R}_{r-1}\rangle$ to obtain the result $|{R}_{r}\rangle$ and sends it to the participant ${P}_{r+1}$. Until ${P}_{t}$’s computational task is completed, the particle $|{R}_{t}\rangle$ is sent to the server, corresponding to step ⑥–⑪ in the Fig. 1.

### Result output stage

The server performs a Lagrange unitary operation on the received particle, and measures the transformed particle to obtain the summation result of multiple participants. This stage consists of the following two steps:

Step 1. The server computes $${\theta }_{v{\prime} }=\frac{2\pi {a}_{0}}{d}\,mod\,2\pi $$, and then performs a Lagrange unitary operation $$m$$$$(\,-\,{\theta }_{v{\prime} })$$ on $$|{R}_{t}\rangle $$ to obtain the particle $$|R\rangle $$, corresponding to step ⑫–⑭ in the Fig. 1.

Step 2. The server measures the particle $|R\rangle$ to obtain the summation result of multiple participants and sends it to all participants via a secure channel.

**Figure 2:**
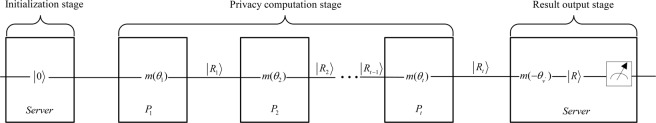
The circuit diagram of the proposed protocol.

A quantum circuit diagram is drawn to describe the execution process of the proposed protocol, as shown in Fig. 2. Here, we omit the shares distribution processes.

In Fig. 2, the server prepares an initial particle, and sends it to the first participant ${P}_{1}$. Then, ${P}_{1}$ performs the Lagrange unitary operation *m*(*θ*_1_) $({\theta }_{1}={\theta }_{{u}_{1}}\,\ast \,{z}_{1}+{\theta }_{{v}_{1}})$ on the received particle to obtain $|{R}_{1}\rangle$, and then sends it to the next participant. Until the last participant completes his computational task, the particle $|{R}_{t}\rangle$ is sent to the server. Finally, the server performs the Lagrange unitary operation *m*$(\,-\,{\theta }_{v{\prime} })$ on the received particle, and then measures the transformed particle to obtain the summation result of multiple participants.

## Correctness Proof

### Theorem 1.

Suppose that $m$(${\theta }_{1}$), $m$(${\theta }_{2}$) are any two $d$-dimensional Lagrange unitary operators. They have the following properties: $m({\theta }_{1})m({\theta }_{2})=m({\theta }_{1}+{\theta }_{2}),m({\theta }_{1})m(-{\theta }_{1})=u$, where $u$ is the unit matrix. The proof process is shown in literature^34^.

### Lemma 1.

According to Theorem 1, when each participant honestly follows the steps in the proposed protocol, the correct collaborative computation result can be obtained ultimately.

### Proof.

In the multiparty privacy computation stage, after each participant has performed the Lagrange unitary operation $m(({\theta }_{{u}_{r}}\ast {z}_{r})+{\theta }_{{v}_{r}})$ on the received particle $|{R}_{r-1}\rangle$, the Eq. (7) can be obtained

7 $$m(({\theta }_{{u}_{t}}\ast {z}_{t})+{\theta }_{{v}_{t}})\cdots m(({\theta }_{{u}_{2}}\ast {z}_{2})+{\theta }_{{v}_{2}})m(({\theta }_{{u}_{1}}\ast {z}_{1})+{\theta }_{{v}_{1}})|0\rangle =|{R}_{t}\rangle .$$

According to Theorem 1, the Eq. (7) can be rewritten as:

8 $$\begin{array}{c}m(({\theta }_{{u}_{t}}\ast {z}_{t})+{\theta }_{{v}_{t}})\cdots m(({\theta }_{{u}_{2}}\ast {z}_{2})+{\theta }_{{v}_{2}})m(({\theta }_{{u}_{1}}\ast {z}_{1})+{\theta }_{{v}_{1}})|0\rangle \\ \,=\,m(\mathop{\sum }\limits_{r=1}^{t}(({\theta }_{{u}_{r}}\ast {z}_{r})+{\theta }_{{v}_{r}}))|0\rangle \\ \,=\,|{R}_{t}\rangle ,\end{array}$$

where ${\sum }_{r=1}^{t}({\theta }_{{u}_{r}}\ast {z}_{r})$ is the sum of private information of all participants, and ${\sum }_{r=1}^{t}\,{\theta }_{{v}_{r}}$ is the sum of share angle of all participants. Therefore, in the multiparty privacy computation stage, $t$ participant’s private information and their share angle are correctly embedded in the phase of a particle.

In the result output stage, the server computes ${\theta }_{v{\prime} }=\frac{2\pi {a}_{0}}{d}\,mod\,2\pi$ with his own secret message ${a}_{0}$. According to $({k}_{1}+{k}_{2}+\cdots +{k}_{t})mod\,d={a}_{0}$, ${\sum }_{r\mathrm{=1}}^{t}\,{\theta }_{{v}_{r}}mod\,2\pi ={\theta }_{v{\prime} }$ can be obtained. Thus, when the server performs *m*$(\,-\,{\theta }_{v{\prime} })$ on $|{R}_{t}\rangle$ to obtain $|R\rangle$, the Eq. (8) can be rewritten as

9 $$m(\,-\,{\theta }_{v{\prime} })|{R}_{t}\rangle =m(\mathop{\sum }\limits_{r=1}^{t}(({\theta }_{{u}_{r}}\ast {z}_{r})+{\theta }_{{v}_{r}})-{\theta }_{v{\prime} })|0\rangle =m(\mathop{\sum }\limits_{r=1}^{t}({\theta }_{{u}_{r}}\ast {z}_{r}))|0\rangle =|R\rangle $$

When all participants honestly perform the proposed protocol steps, the correctness of the proposed protocol can be proved.

## Security Analysis

In this section, the security of the proposed protocol is analyzed from five aspects: intercept-measurement attack, intercept-resend attack, entangle-swapping attack, entangle-measurement attack and collusion attack.

### Intercept-measurement attack

Suppose that there is a malicious attacker Eve who wants to perform interception-measurement attack among $t$ participants. When participant ${P}_{r}(r\in \{1,2,\ldots ,t\})$ completes his calculation task and sends the calculation result $|{R}_{r}\rangle$ to the next participant ${P}_{r+1}$, Eve intercepts the calculation result. Then, Eve tries to measure the particle $|{R}_{r}\rangle$ and steal the privacy information $({\theta }_{{u}_{r}}\ast {z}_{r})$ of participant ${P}_{r}$.

For example, in the multi-party privacy computation stage of the proposed protocol, the attacker Eve wants to obtain the privacy information $({\theta }_{{u}_{1}}\ast {z}_{1})$ of the participant ${P}_{1}$ through interception-measurement attack. Suppose that the participant ${P}_{1}$ completes his calculation task, when he sends the calculation result $|{R}_{1}\rangle$ to the next participant ${P}_{2}$, Eve intercepts the particle $|{R}_{1}\rangle$ on the transmission route from ${P}_{1}$ to ${P}_{2}$. She measures the particle by using the base $\{m(\theta )|0\rangle |\theta =0,\frac{2\pi }{d},\ldots ,\frac{(d-1)2\pi }{d}\}$ to obtain $(({\theta }_{{u}_{1}}\ast {z}_{1})+{\theta }_{{v}_{1}})$. The value of ${\theta }_{{v}_{1}}$ is computed by ${P}_{1}$’s privacy share $f({x}_{1})$, however, Eve does not know $f({x}_{1})$. Even if Eve obtains $({\theta }_{{u}_{1}}\ast {z}_{1})+{\theta }_{{v}_{1}}$, she cannot deduce the value of the privacy information $({\theta }_{{u}_{1}}\ast {z}_{1})$ from it.

When Eve wants to obtain the private information $({\theta }_{{u}_{h}}\ast {z}_{h})$ of participant ${P}_{h}(h\in \{2,3,\ldots ,t\})$, she will fail. For example, suppose that the participant ${P}_{h}$ completes his calculation task, when he sends the calculation result $|{R}_{h}\rangle$ to the next participant ${P}_{h+1}$, Eve intercepts the particles on the transmission route from ${P}_{h}$ to ${P}_{h+1}$. She measures the particle by using the base $\{m(\theta )|0\rangle |\theta =0,\frac{2\pi }{d},\ldots ,\frac{(d-1)2\pi }{d}\}$ to obtain ${\sum }_{i=1}^{h}(({\theta }_{{u}_{i}}\,\ast \,{z}_{i})+{\theta }_{{v}_{i}})$. The result ${\sum }_{i=1}^{h}(({\theta }_{{u}_{i}}\ast {z}_{i})+{\theta }_{{v}_{i}})$ is the sum of the private information of the first participant to the $h$ participant, she cannot deduce the private information $({\theta }_{{u}_{h}}\ast {z}_{h})$ of the participant ${P}_{h}$ from it. Thus, the proposed protocol can resist intercept-measurement attack.

### Intercept-resend attack

Suppose that there is a malicious attacker Eve who wants to perform an interception-resend attack among $t$ participants. When participant ${P}_{r}(r\in \{1,2,\ldots ,t\})$ completes his calculation task and sends the calculation result $|{R}_{r}\rangle$ to the next participant ${P}_{r+1}$, Eve intercepts the calculation result. Then, Eve prepares a new particle and sends it to the next participant ${P}_{r+1}$. After ${P}_{r+1}$ completes the operation task and sends the particle to ${P}_{r+2}$, Eve intercepts the particle and try to steal the privacy information $({\theta }_{{u}_{r+1}}\ast {z}_{r+1})$ of participant ${P}_{r+1}$.

For example, in the multiparty privacy computation stage of the proposed protocol, suppose that the participant ${P}_{1}$ completes his calculation task, when he sends the calculation result $|{R}_{1}\rangle$ to the next participant ${P}_{2}$, Eve intercepts the particle $|{R}_{1}\rangle$ on the transmission route from ${P}_{1}$ to ${P}_{2}$. Then, she prepares a new particle $|0\rangle$ and sends it to ${P}_{2}$. Suppose that the participant ${P}_{2}$ completes his calculation task, when he sends the calculation result $|{R}_{2}\rangle$ to the next participant ${P}_{3}$, Eve intercepts the particle $|{R}_{2}\rangle$ on the transmission route from ${P}_{2}$ to ${P}_{3}$. She measures the particle by using the base $\{m(\theta )|0\rangle |\theta =0,\frac{2\pi }{d},\ldots ,\frac{(d-1)2\pi }{d}\}$ to obtain $(({\theta }_{{u}_{2}}\ast {z}_{2})+{\theta }_{{v}_{2}})$. Since the attacker does not know the privacy share $f({x}_{2})$, she cannot obtain the value of ${\theta }_{{v}_{2}}$, let alone the privacy information $({\theta }_{{u}_{2}}\ast {z}_{2})$ of the participant ${P}_{2}$.

### Entanglement-swapping attack

The entanglement-swapping is a joint measurement, which is to swapping entanglement states by measuring between different particles. This requires that the particle containing the privacy information is a multi-particle entangled state. But in the proposed protocol, a single particle is used for privacy information storage, not an entangled particles. Thus, the proposed protocol can resist entanglement-swapping attacks.

### Entanglement-measurement attack

Suppose that the attacker Eve attempts to carry out an entanglement-measurement attack among $t$ participants. Suppose that the participant ${P}_{r}(r\in \{1,2,\ldots ,t\})$ completes his calculation task, when he sends the calculation result $|{R}_{r}\rangle$ to the next participant ${P}_{r+1}$, Eve intercepts the calculation result $|{R}_{r}\rangle$ on the transmission route from ${P}_{r}$ to ${P}_{r+1}$. Then, she prepares an ancilla particle and performs the $CNOT$ operation on the intercepted particle and the ancilla particle to generate a 2-particle entangled state. Finally, Eve measures the ancilla particle to obtain the privacy information $({\theta }_{{u}_{r}}\ast {z}_{r})$ of participant ${P}_{r}$.

For example, the attacker Eve wants to obtain the privacy information $({\theta }_{{u}_{1}}\ast {z}_{1})$ of the participant ${P}_{1}$. suppose that the participant ${P}_{1}$ completes his calculation task, when he sends the calculation result $|{R}_{1}\rangle$ to the next participant ${P}_{2}$, Eve intercepts the particle $|{R}_{1}\rangle$ on the transmission route from ${P}_{1}$ to ${P}_{2}$. Then, she prepares a $d$-dimensional ancilla particle $|c\rangle (c\in \{0,1,\ldots ,d-1\})$ and uses $|{R}_{1}\rangle$ as the control particle and $|c\rangle$ as the target particle to perform $CNOT$ operation to obtain $|{R}_{1}\rangle |c+{R}_{1}\rangle$. Eve measures the ancilla particle $|c+{R}_{1}\rangle$ to obtaion $c+{R}_{1}$ and deduce ${R}_{1}$ from it. She can obtain $({\theta }_{{u}_{1}}\ast {z}_{1})+{\theta }_{{v}_{1}}$ of the participant ${P}_{1}$ by the base $\{m(\theta )|0\rangle |\theta =0,\frac{2\pi }{d},\ldots ,\frac{(d-1)2\pi }{d}\}$. But ${\theta }_{{v}_{1}}$ is computed by $f({x}_{1})$, Eve does not know the privacy share $f({x}_{1})$. Even if Eve obtains $({\theta }_{{u}_{1}}\ast {z}_{1})+{\theta }_{{v}_{1}}$, she cannot deduce the privacy information $({\theta }_{{u}_{1}}\ast {z}_{1})$ from it. Thus, the proposed protocol can resist entanglement-measurement attack.

### Collusion attack

In privacy computation stage, suppose that $t$ participants want to carry out collaborative computing, in which $c(1 < c < t)$ participants want to collude and obtain privacy information $({\theta }_{u}\ast z)$ of other participants $\{{P}_{1},{P}_{2},\ldots ,{P}_{t-c}\}$.

For example, suppose that there are 4 participants $\{{P}_{1},{P}_{2},{P}_{3},{P}_{4}\}$ want to carry out collaborative computation, in which 2 participants *P*_1_, *P*_3_ want to collude and obtain privacy information $({\theta }_{{u}_{2}}\ast {z}_{2})$ of other 1 participant ${P}_{2}$. the colluder ${P}_{1}$ records the information of $({\theta }_{{u}_{1}}\ast {z}_{1})+{\theta }_{{v}_{1}}$ after the operation task is completed, then he sends the particle $|{R}_{1}\rangle$ to the next participant ${P}_{2}$. After ${P}_{2}$ operation task is completed, ${P}_{2}$ sends the particle $|{R}_{2}\rangle$ to ${P}_{3}$. After the colluder ${P}_{3}$ receiving the particle, he records the information of $({\theta }_{{u}_{1}}\ast {z}_{1})+{\theta }_{{v}_{1}}$ from ${P}_{1}$. Then, ${P}_{3}$ performs $m(\,-\,(({\theta }_{{u}_{1}}\ast {z}_{1})+{\theta }_{{v}_{1}}))$ on the particle $|{R}_{2}\rangle$ to obtain a particle that contains the privacy information of the participant ${P}_{2}$. ${P}_{3}$ measures the particle by using the base $\{m(\theta )|0\rangle |\theta =0,\frac{2\pi }{d},\ldots ,\frac{(d-1)2\pi }{d}\}$ to obtain $({\theta }_{{u}_{2}}\ast {z}_{2})+{\theta }_{{v}_{2}}$ of the participant ${P}_{2}$. ${\theta }_{{v}_{2}}$ is computed by the participant ${P}_{2}$’s privacy share $f({x}_{2})$, however, ${P}_{1},{P}_{3}$ does not know $f({x}_{2})$. Even if ${P}_{3}$ obtains $({\theta }_{{u}_{2}}\ast {z}_{2})+{\theta }_{{v}_{2}}$, he can not deduce the privacy information $({\theta }_{{u}_{2}}\ast {z}_{2})$ from it.

In the result output stage, after $4$ participants receive the calculation results, the participants *P*_1_, *P*_3_ want to collude and obtain private information $({\theta }_{u}\ast z)$ of other 2 participants *P*_2_, *P*_4_. Because the calculation result is calculated by the privacy information of four participants, even if participants ${P}_{1}$ and ${P}_{3}$ collude, they can only obtain the sum of the privacy information of the participants ${P}_{2}$ and ${P}_{4}$, and they cannot deduce the private information of any one participant. Thus, the proposed protocol can resist collusion attack.

## Simulation

In this section, the proposed protocol is simulated by a specific example on the classical computer. The simulation mainly focouses on the privacy computation stage and result output stage.

Suppose that all quantum states are on 7-dimensional Hilbert space. In the proposed protocol, there are 5 participants ${P}_{1},{P}_{2},{P}_{3},{P}_{4},{P}_{5}$, where $t=3,n=5,d=7$. The server randomly chooses 3 privacy integers ${a}_{0}=5,{a}_{1}=3,{a}_{2}=2$ and 5 public distinct and no zero integers ${x}_{1}=1,{x}_{2}=2,{x}_{3}=3,{x}_{4}=5,{x}_{5}=6$, where ${a}_{0}=5$ is a secret message. The server first constructs a polynomial with degree 2: $f(x)=2{x}^{2}+3x+5\,{\rm{mod}}\,7$, and then he generates 5 secret shares $f({x}_{1})=3,f({x}_{2})=5,f({x}_{3})=4,f({x}_{4})=0,f({x}_{5})=4$. Further, the server distributes each secret share $f$(${x}_{r}$)($r=1,2,\ldots ,5$) to the corresponding participant via a secure channel. Now three participants ${P}_{1},{P}_{2},{P}_{3}$ want to joint computation, and the parameter one ${\theta }_{u}$ of the three participants are ${\theta }_{{u}_{1}}=1.79519,{\theta }_{{u}_{2}}=0,{\theta }_{{u}_{3}}=2.69279$, the parameter two $z$ of the three participants are ${z}_{1}=1,{z}_{2}=4,{z}_{3}=2$. The Lagrange unitary operator $m(\theta )$ on the 7-dimensional Hilbert space is shown in appendix.

As can be see from Table 1, the participant ${P}_{1}$ first calculates out the secret shadow ${k}_{1}=f({x}_{1}){\prod }_{1\le m\le t,m\ne 1}\frac{{x}_{m}}{{x}_{m}-{x}_{1}}\,mod\,d=2$ and ${\theta }_{{v}_{1}}=\frac{2\pi {k}_{1}}{d}\,mod\,2\pi =1.79519$. Further ${P}_{1}$ performs Lagrange unitary operation $m(({\theta }_{{u}_{1}}\,\ast \,{z}_{1})+{\theta }_{{v}_{1}})$ on $|{R}_{0}\rangle =|0\rangle$ to obtain the particle $|{R}_{1}\rangle$ and sends it to the the participant ${P}_{2}$. Untill participants ${P}_{2}$, ${P}_{3}$ have completed their computational tasks, the private information of three participants have been embedded into the particle $|{R}_{3}\rangle$.

**Table 1:** Simulation processes of multiparty privacy computation stage.

Participant	Received particle |*R*_*r*−1_〉	Parameter one ${{\boldsymbol{\theta }}}_{{{\boldsymbol{u}}}_{{\boldsymbol{r}}}}$	Parameter two *z*_*r*_	Secret share *f*(*x*_*r*_)	Secret shadow *k*_*r*_	Secret angle ${{\boldsymbol{\theta }}}_{{{\boldsymbol{v}}}_{{\boldsymbol{r}}}}$	Participant operation results |*R*_*r*_〉
*P*_1_	|0〉	_1.79519_	1	3	2	1.79519	$[[1.15799\times {10}^{-16}-1.28760\times {10}^{-16}i]$
							$$[6.04340\times {10}^{-17}-2.07313\times {10}^{-16}i]$$
							$$[\,-\,6.3970\times {10}^{-17}-3.8382\times {10}^{-16}i]$$
							$$[1.0000\times {10}^{0}+3.42951\times {10}^{-16}i]$$
							$$[3.39967\times {10}^{-16}+1.89294\times {10}^{-16}i]$$
							$$[2.15563\times {10}^{-16}+1.27872\times {10}^{-17}i]$$
							$$[1.60198\times {10}^{-16}-6.57657\times {10}^{-17}i]]$$
*P*_2_	$[[1.15799\times {10}^{-16}-1.28760\times {10}^{-16}i]$	_0.00000_	4	5	6	5.38558	$[[3.65398\times {10}^{-16}-1.64584\times {10}^{-16}i]$
	$[6.04340\times {10}^{-17}-2.07313\times {10}^{-16}i]$						$[2.57801\times {10}^{-16}-3.06826\times {10}^{-16}i]$
	$[\,-\,6.3970\times {10}^{-17}-3.8382\times {10}^{-16}i]$						$[1.23631\times {10}^{-16}-4.84198\times {10}^{-16}i]$
	$[1.0000\times {10}^{0}+3.42951\times {10}^{-16}i]$						$[\,-\,1.77847\times {10}^{-16}-8.82750\times {10}^{-16}i]$
	$[3.39967\times {10}^{-16}+1.89294\times {10}^{-16}i]$						$[1.00000\times {10}^{0}+1.23575\times {10}^{-15}i]$
	$[2.15563\times {10}^{-16}+1.27872\times {10}^{-17}i]$						$[8.01047\times {10}^{-16}+4.11338\times {10}^{-16}i]$
	$[1.60198\times {10}^{-16}-6.57657\times {10}^{-17}i]]$						$[4.99569\times {10}^{-16}+1.27872\times {10}^{-17}i]]$
*P*_3_	$[[3.65398\times {10}^{-16}-1.64584\times {10}^{-16}i]$	_2.69279_	2	4	4	_3.59039_	$[[\,-\,1.57589\times {10}^{-16}-1.06254\times {10}^{-15}i]$
	$[2.57801\times {10}^{-16}-3.06826\times {10}^{-16}i]$						$[1.00000\times {10}^{0}+1.46439\times {10}^{-15}i]$
	$[1.23631\times {10}^{-16}-4.84198\times {10}^{-16}i]$						$[9.28988\times {10}^{-16}+5.39279\times {10}^{-16}i]$
	$[\,-\,1.77847\times {10}^{-16}-8.82750\times {10}^{-16}i]$						$[5.94345\times {10}^{-16}+4.59509\times {10}^{-17}i]$
	$[1.00000\times {10}^{0}+1.23575\times {10}^{-15}i]$						$[4.45415\times {10}^{-16}-1.73600\times {10}^{-16}i]$
	$[8.01047\times {10}^{-16}+4.11338\times {10}^{-16}i]$						$[3.25983\times {10}^{-16}-3.49667\times {10}^{-16}i]$
	$[4.99569\times {10}^{-16}+1.27872\times {10}^{-17}i]]$						$[1.77053\times {10}^{-16}-5.69219\times {10}^{-16}i]]$

In result output stage, the server uses the secret message ${a}_{0}$ to perform Lagrange unitary operation on the received particle $|{R}_{3}\rangle$. According to Shamir ($t,n$) threshold scheme, Owing to $({k}_{1}+{k}_{2}+{k}_{3})\,mod\,d={a}_{0}$ in the Shamir threshold scheme, ${\sum }_{r=1}^{3}\,{\theta }_{{v}_{r}}\,mod\,2\pi =-\,{\theta }_{v{\prime} }$ can be obtained. Thus, when the server performs *m*$(\,-\,{\theta }_{v{\prime} })$ on $|{R}_{3}\rangle$, he can obtain $|R\rangle$.

$$\begin{array}{rcl}m(\,-\,{\theta }_{v{\prime} })|{R}_{3}\rangle  & = & m(\,-\,4.48798)(\begin{array}{c}\,-\,1.57589\times {10}^{-16}-1.06254\times {10}^{-15}i\\ 1.00000\times {10}^{0}+1.46439\times {10}^{-15}i\\ 9.28988\times {10}^{-16}+5.39279\times {10}^{-16}i\\ 5.94345\times {10}^{-16}+4.59509\times {10}^{-17}i\\ 4.45415\times {10}^{-16}-1.73600\times {10}^{-16}i\\ 3.25983\times {10}^{-16}-3.49667\times {10}^{-16}i\\ 1.77053\times {10}^{-16}-5.69219\times {10}^{-16}i\end{array})\\  & = & (\begin{array}{c}1.05877\times {10}^{-15}+5.99422\times {10}^{-16}i\\ 6.73718\times {10}^{-16}+4.47723\times {10}^{-17}i\\ 5.02354\times {10}^{-16}-2.02070\times {10}^{-16}i\\ 3.64930\times {10}^{-16}-4.00022\times {10}^{-16}i\\ 1.93566\times {10}^{-16}-6.46864\times {10}^{-16}i\\ \,-\,1.91486\times {10}^{-16}-1.20151\times {10}^{-15}i\\ 1.00000\times {10}^{0}+1.52624\times {10}^{-15}i\end{array})\\  & = & |R\rangle .\end{array}$$

In order to verify the correctness of the simulation results, we performs the Lagrange unitary operator with the private information $({\theta }_{{u}_{r}}\ast {z}_{r})$ ($r=1,2,3$) on particle $|0\rangle$ to obtain result $|R{\prime} \rangle$. We compare $|R\rangle$ and $|R{\prime} \rangle$ to judge whether the result of the collaborative computation is correct or not.

$$\begin{array}{rcl}m(\mathop{\sum }\limits_{i=1}^{3}({\theta }_{{u}_{i}}\ast {z}_{i}))|0\rangle  & = & m((1\ast 1.79519)+(4\ast 0.00000)+(2\ast 2.69279))|0\rangle \\  & = & (\begin{array}{c}1.05877\times {10}^{-15}+5.99422\times {10}^{-16}i\\ 6.73718\times {10}^{-16}+4.47723\times {10}^{-17}i\\ 5.02354\times {10}^{-16}-2.02070\times {10}^{-16}i\\ 3.64930\times {10}^{-16}-4.00022\times {10}^{-16}i\\ 1.93566\times {10}^{-16}-6.46864\times {10}^{-16}i\\ \,-\,1.91486\times {10}^{-16}-1.20151\times {10}^{-15}i\\ 1.00000\times {10}^{0}+1.52624\times {10}^{-15}i\end{array})\\  & = & |R{\prime} \rangle .\end{array}$$

From the above calculation results, the particle $|R\rangle$ is the same as the particle $|R{\prime} \rangle$. Thus, the results of the collaborative computation of the three participants are correct.

## Performance Analysis and Comparison

In this section, the performance of the proposed protocol is analyzed and compared with the three other similar protocols(refs. ^31,33^ and the first protocol of multiparty summation computation in ref. ^32^). We suppose that all protocols have $n$ participants and when ref. ^32^ is compared to the proposed protocol, all quantum states are on $2$-dimensional Hilbert space. When ref. ^31^ and^33^ is compared with the proposed protocol, all quantum states are on *d*-dimensional Hilbert space. We can be viewed from the following seven aspects: Space dimension, Number of initial particles, Number of entangled states, Number of $QFT$ operations, Number of unitary operations, Number of $QF{T}^{-1}$ operations, Number of measurement operations.

### Space dimension

It can be see from Table 2, ref. ^32^ is a 2-dimensional protocol and the other references are *d*-dimensional protocols on the Hilbert space.

**Table 2:** Comparison of the four protocols.

	Ref. ^31^	Ref. ^32^	Ref. ^33^	The proposed protocol
Space dimension	*d*	2	*d*	*d*
Number of initial particles	*n* + 1	1	*n*^2^	1
Number of entangled states	1	0	*n*	0
Number of *QFT* operations	1	0	*n*^2^	0
Number of unitary operations	*n*	2*n* + 1	*n*^2^	*n* + 1
Number of *QFT*^−1^ operations	1	0	0	0
Number of measurement operations	0	1	*n*^2^	1

### Number of initial particles

In ref. ^31^, the first participant prepares two initial particle and the other participants prepare an initial particle, so the number of initial particles in ref. ^31^ is $n+1$. In ref. ^32^ and the proposed protocol, the server prepares an initial particle to carry privacy information of all participants, so the number of particles in ref. ^32^ and the proposed protocol are 1. In ref. ^33^, the first participant prepares $n$ entangled states, each of which has $n$ particle, so the number of initial particles in ref. ^33^ is *n*^2^.

### Number of entangled states

In ref. ^31^, the first participant performs the $QFT$ and $CNOT$ operations on the two initial particle to generate a 2-particle entangled state, so the number of entangled states in ref. ^31^ is 1. In ref. ^33^, the first participant prepares $n$ entangled states, each of which has $n$ particles, so the number of entangled states in ref. ^33^ is $n$. In ref. ^32^ and the proposed protocol, entangled states is not being used, so the number of entangled states in ref. ^32^ and the proposed protocol are 0.

### Number of *QFT* operations

In ref. ^31^, the first participant performs one $QFT$ operations on two particle to prepare an entangled state, so the number of $QFT$ operations in ref. ^31^ is 1. In ref. ^33^, each participant has $n$ privacy data and receives $n$ quantum sequence from dealer, and then he embeds the $n$ privacy data in the received quantum sequence by performing $QFT$ operations and unitary operations, so the number of $QFT$ operations in ref. ^33^ is $n$. In ref. ^32^ and the proposed protocol, $QFT$ operations is not being used, so the number of $QFT$ operations in ref. ^32^ and the proposed protocol are 0.

### Number of unitary operations

In ref. ^31^, the previous participant sends a particle of the entangled state to the next participant. After the current participant receives the particle, a new particle containing the private information is prepared. He performs the unitary operations on the received particle and the prepared particle. The transformed particle is sent to the next participant until all participants have completed their operational task. Thus, the number of unitary operations in ref. ^31^ is $n$. In ref. ^32^, the server prepares a particle send it to the first participant. The first participant performs two unitary operations on the received particle to embed the privacy input and the secret input into the phase of the particle. He sends the transformed particle to the next participant, untill all participant have completed her computational tasks. Thus, the number of unitary operations in ref. ^32^ is $2n+1$. In ref. ^33^, each participant has $n$ privacy data and receives $n$ quantum sequence from dealer. Each participant performs $QFT$ and unitary operations on $n$ particles of entangled states to embed their own $n$ privacy data into the phases of $n$ particles. Thus, the number of unitary operations in ref. ^33^ is ${n}^{2}$. In the proposed protocol, all participants and the server performs the Lagrange unitary operation on the particle, so the number of unitary operations in the proposed protocol is $n+1$.

### Number of *QFT*^−1^ operations

In ref. ^31^, after all participants have completed their computation tasks, the first participant uses the $QF{T}^{-1}$ operations to obtain the sum of the privacy data of all participants, so the number of $QF{T}^{-1}$ operation in ref. ^31^ is $1$. In refs. ^32,33^ and the proposed protocol, $QF{T}^{-1}$ operations is not being used, so the number of $QF{T}^{-1}$ in refs ^32,33^ are $0$.

### Number of measurement operations

In ref. ^32^ and the proposed protocol, the server measures a particle to obtain the computation result, so the number of measurement operations in ref. ^32^ and the proposed protocol are $1$. In ref. ^33^, each participant measures particle of the entangled states to obtain the computation result, so the number of measurement operations in ref. ^33^ is ${n}^{2}$. In ref. ^31^, the measurement operations is not being used, so the number of measurement operations in ref. ^31^ is 0.

### Summary

From the performance analysis and comparison as mentioned above, we draw conclusions from three aspects: universality and practicability, computation cost, resource cost.

Compared the ref. ^32^ with the proposed protocol, the ref. ^32^ is a 2-dimensional protocol and the proposed protocol is $d$-dimensional protocols on the Hilbert space, so the proposed protocol has better universality and practicality. In the computation efficiency aspect, the total number of $QFT$ operations, $QF{T}^{-1}$ operations and number of unitary operations in the proposed protocol is smaller than that in ref. ^32^, so it has higher computation efficiency. In the resource consumption cost aspect, the total number of initial particles, entangled states and number of measurement operations in the proposed protocol is same as that in ref. ^32^, so they have same resource consumption cost.

**Figure 3:**
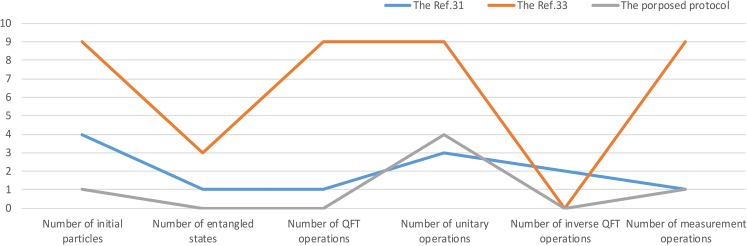
The flow chart of performance comparison.

Compared the refs. ^31,33^ with the proposed protocol, suppose that the number of participants *n* = 3, and the dimensions of Hilbert space, the three protocols are same. In the three protocols, the numbers of initial particles are 4, 9, 1, respectively, and the numbers of entangled states are 1, 3, 0, respectively, and the numbers of $QFT$ operations are 1, 9, 0, respectively, and the numbers of unitary operations are 3, 9, 4, respectively, and the numbers of $QF{T}^{-1}$ operations are 1, 0, 0, respectively, and the numbers of measurement operations are 0, 9, 1, respectively. As shown in Fig. 3, in the computational efficiency aspect, the number of $QFT$ operations, the number of $QF{T}^{-1}$ operations and the number of unitary operations in the proposed protocol is the same as that in ref. ^31^, but higher than that in refs. ^33^. In the resource consumption cost aspect, the number of initial particles, the number of entangled states and the number of measurement operations in the proposed protocol is smaller than that in refs. ^31^ and^33^, so it has lower resource consumption cost.

## Conclusion

In this paper, we propose a secure multiparty quantum computation protocol based on Lagrange unitary operator, which performs Lagrange unitary operator on the single particle to obtain the summation of the computational results of multiple participants. In addition, the Shamir $(t,n)$ threshold scheme is employed to the proposed protocol to ensure its security. The security analysis shows that the proposed protocol can resist interception-measurement attack, intercept-resend attack, entanglement-swapping attack, entanglement-measurement attack, collusion attack. The simulation experiment proves the correctness of the result of the proposed protocol. Compared with other existing similar protocols, the proposed protocol has lower resource consumption cost and higher computational efficiency.

## Appendix

When *q* is 7 × 7 matrix, the set $\{{q}^{0},{q}^{1},{q}^{2},{q}^{3},{q}^{4},{q}^{5},{q}^{6}\}$ is defined as follows

$$\begin{array}{ll}{q}^{0}=(\begin{array}{ccccccc}1 & 0 & 0 & 0 & 0 & 0 & 0\\ 0 & 1 & 0 & 0 & 0 & 0 & 0\\ 0 & 0 & 1 & 0 & 0 & 0 & 0\\ 0 & 0 & 0 & 1 & 0 & 0 & 0\\ 0 & 0 & 0 & 0 & 1 & 0 & 0\\ 0 & 0 & 0 & 0 & 0 & 1 & 0\\ 0 & 0 & 0 & 0 & 0 & 0 & 1\end{array}), & {q}^{1}=(\begin{array}{ccccccc}0 & 1 & 0 & 0 & 0 & 0 & 0\\ 0 & 0 & 1 & 0 & 0 & 0 & 0\\ 0 & 0 & 0 & 1 & 0 & 0 & 0\\ 0 & 0 & 0 & 0 & 1 & 0 & 0\\ 0 & 0 & 0 & 0 & 0 & 1 & 0\\ 0 & 0 & 0 & 0 & 0 & 0 & 1\\ 1 & 0 & 0 & 0 & 0 & 0 & 0\end{array}),\\ {q}^{2}=(\begin{array}{ccccccc}0 & 0 & 1 & 0 & 0 & 0 & 0\\ 0 & 0 & 0 & 1 & 0 & 0 & 0\\ 0 & 0 & 0 & 0 & 1 & 0 & 0\\ 0 & 0 & 0 & 0 & 0 & 1 & 0\\ 0 & 0 & 0 & 0 & 0 & 0 & 1\\ 1 & 0 & 0 & 0 & 0 & 0 & 0\\ 0 & 1 & 0 & 0 & 0 & 0 & 0\end{array}), & {q}^{3}=(\begin{array}{ccccccc}0 & 0 & 0 & 1 & 0 & 0 & 0\\ 0 & 0 & 0 & 0 & 1 & 0 & 0\\ 0 & 0 & 0 & 0 & 0 & 1 & 0\\ 0 & 0 & 0 & 0 & 0 & 0 & 1\\ 1 & 0 & 0 & 0 & 0 & 0 & 0\\ 0 & 1 & 0 & 0 & 0 & 0 & 0\\ 0 & 0 & 1 & 0 & 0 & 0 & 0\end{array}),\\ {q}^{4}=(\begin{array}{ccccccc}0 & 0 & 0 & 0 & 1 & 0 & 0\\ 0 & 0 & 0 & 0 & 0 & 1 & 0\\ 0 & 0 & 0 & 0 & 0 & 0 & 1\\ 1 & 0 & 0 & 0 & 0 & 0 & 0\\ 0 & 1 & 0 & 0 & 0 & 0 & 0\\ 0 & 0 & 1 & 0 & 0 & 0 & 0\\ 0 & 0 & 0 & 1 & 0 & 0 & 0\end{array}), & {q}^{5}=(\begin{array}{ccccccc}0 & 0 & 0 & 0 & 0 & 1 & 0\\ 0 & 0 & 0 & 0 & 0 & 0 & 1\\ 1 & 0 & 0 & 0 & 0 & 0 & 0\\ 0 & 1 & 0 & 0 & 0 & 0 & 0\\ 0 & 0 & 1 & 0 & 0 & 0 & 0\\ 0 & 0 & 0 & 1 & 0 & 0 & 0\\ 0 & 0 & 0 & 0 & 1 & 0 & 0\end{array}),\\ {q}^{6}=(\begin{array}{ccccccc}0 & 0 & 0 & 0 & 0 & 0 & 1\\ 1 & 0 & 0 & 0 & 0 & 0 & 0\\ 0 & 1 & 0 & 0 & 0 & 0 & 0\\ 0 & 0 & 1 & 0 & 0 & 0 & 0\\ 0 & 0 & 0 & 1 & 0 & 0 & 0\\ 0 & 0 & 0 & 0 & 1 & 0 & 0\\ 0 & 0 & 0 & 0 & 0 & 1 & 0\end{array}). & \end{array}$$

Based on Eq. (2), we can obtain Lagrange unitary operator *m*(*θ*) as follows

$$\begin{array}{rcl}m(\theta ) & = & \left(\frac{{\prod }_{k\ne 0}({e}^{i\theta }-{\omega }^{k})}{{\prod }_{k\ne 0}({\omega }^{0}-{\omega }^{k})}\right){q}^{0}+\left(\frac{{\prod }_{k\ne 1}({e}^{i\theta }-{\omega }^{k})}{{\prod }_{k\ne 1}({\omega }^{1}-{\omega }^{k})}\right){q}^{1}\\  &  & +\,\left(\frac{{\prod }_{k\ne 2}({e}^{i\theta }-{\omega }^{k})}{{\prod }_{k\ne 2}({\omega }^{2}-{\omega }^{k})}\right){q}^{2}+\left(\frac{{\prod }_{k\ne 3}({e}^{i\theta }-{\omega }^{k})}{{\prod }_{k\ne 3}({\omega }^{3}-{\omega }^{k})}\right){q}^{3}\\  &  & +\,\left(\frac{{\prod }_{k\ne 4}({e}^{i\theta }-{\omega }^{k})}{{\prod }_{k\ne 4}({\omega }^{4}-{\omega }^{k})}\right){q}^{4}+\left(\frac{{\prod }_{k\ne 5}({e}^{i\theta }-{\omega }^{k})}{{\prod }_{k\ne 5}({\omega }^{5}-{\omega }^{k})}\right){q}^{5}\\  &  & +\,\left(\frac{{\prod }_{k\ne 6}({e}^{i\theta }-{\omega }^{k})}{{\prod }_{k\ne 6}({\omega }^{6}-{\omega }^{k})}\right){q}^{6},\end{array}$$

where $$\omega ={e}^{i2\pi /7}$$.

## References

[1] Yao, A. C. Protocols for secure computations. In *23rd Annual Symposium on Foundations of Computer Science*, 160–164, 10.1109/SFCS.1982.38 (1982).

[2] Goldreich, O., Micali, S. & Wigderson, A. How to play any mental game. In *Proceedings of the Nineteenth Annual ACM Symposium on Theory of Computing*, 218–229, 10.1145/28395.28420 (ACM, 1987).

[3] Cleve R, Gottesman D, Lo H-K (1999). How to share a quantum secret. Phys. Rev. Lett..

[4] Guo GP, Guo GC (2003). Quantum secret sharing without entanglement. Phys. Lett. A.

[5] Zhang ZJ, Man ZX (2005). Multiparty quantum secret sharing of classical messages based on entanglement swapping. Phys. Rev. A.

[6] Lin S, Gao F, Guo FZ, Wen QY, Zhu FC (2007). Comment on “multiparty quantum secret sharing of classical messages based on entanglement swapping”. Phys. Rev. A.

[7] Zhang KJ, Zhang X, Jia HY, Zhang L (2019). A new n-party quantum secret sharing model based on multiparty entangled states. Quantum Inf. Process..

[8] Jakobi M (2011). Practical private database queries based on a quantum-key-distribution protocol. Phys. Rev. A.

[9] Wei Y, Chun, Wang TY, Gao F (2016). Practical quantum private query with better performance in resisting joint-measurement attack. Phys. Rev. A.

[10] Yang YG, Sun SJ, Xu P, Tian J (2014). Flexible protocol for quantum private query based on b92 protocol. Quantum Inf. Process..

[11] Arrighi P, Salvail L (2008). Blind quantum computation. Int. J. Quantum Inf..

[12] Morimae T, Fujii K (2013). Blind quantum computation protocol in which alice only makes measurements. Phys. Rev. A.

[13] Li Q, Chan WH, Wu CH, Wen ZH (2014). Triple-server blind quantum computation using entanglement swapping. Phys.rev.a.

[14] Wang TY, Yan Wen Q, Gao F, Lin S, Chen Zhu F (2008). Cryptanalysis and improvement of multiparty quantum secret sharing schemes. Phys. Lett. A.

[15] Wang TY, Wen QY (2011). Security of a kind of quantum secret sharing with single photons. Quantum Inf. Computation.

[16] Wang, T. Y., Liu, Y. Z., Wei, C. Y., Cai, X. Q. & Ma, J. F. Security of a kind of quantum secret sharing with entangled states. *Scientific Reports* **7**, 10.1038/s41598-017-02543-0 (2017). 10.1038/s41598-017-02543-0 PMC5449411 28559570

[17] Shi RH, Zhang MW (2019). Privacy-preserving quantum sealed-bid auction based on grover’s search algorithm. Sci. Rep..

[18] Crépeau, C., Gottesman, D. & Smith, A. Secure multi-party quantum computation. In *Proceedings of the Thiry-fourth Annual ACM Symposium on Theory of Computing*, 643–652, 10.1145/509907.510000 (ACM, 2002).

[19] Ben-Or, M., Crepeau, C., Gottesman, D., Hassidim, A. & Smith, A. Secure multiparty quantum computation with (only) a strict honest majority. In *2006 47th Annual IEEE Symposium on Foundations of Computer Science*, 249–260, 10.1109/FOCS.2006.68 (2006).

[20] Damgård, I., Ishai, Y., Krøigaard, M., Nielsen, J. B. & Smith, A. Scalable multiparty computation with nearly optimal work and resilience. In *dvances in Cryptology*, 241–261, 10.1007/978-3-540-85174-5_14 (2008).

[21] Unruh, D. Universally composable quantum multi-party computation. In *Advances in Cryptology*, 486–505, 10.1007/978-3-642-13190-5 (2010).

[22] He LB, Huang LS, Yang W, Xu R (2012). A protocol for the secure two-party quantum scalar product. Phys. Lett. A.

[23] Li YB, Wen QY, Qin SJ (2013). Improved secure multiparty computation with a dishonest majority via quantum means. Int. J. Theor. Phys..

[24] Shi RH (2019). A generic quantum protocol for one-sided secure two-party classical computations. Quantum Inf. Process..

[25] Heinrich S (2002). Quantum summation with an application to integration. J. Complex..

[26] Heinrich, M., Kwas, S. & Woźniakowski, H. Quantum boolean summation with repetitions in the worst-average setting. In *Monte Carlo and Quasi-Monte Carlo Methods 2002*, 243–258, 10.1007/978-3-642-18743-8_14 (2004).

[27] Du JZ, Chen XB, Wen QY, Zhu FC (2007). Secure multiparty quantum summation. China-Phys.

[28] Chen XB, Xu G, Yang YX, Wen QY (2010). An efficient protocol for the secure multi-party quantum summation. Int. J. Theor. Phys..

[29] Zhang C, Sun ZW, Huang Y, Long DY (2014). High-capacity quantum summation with single photons in both polarization and spatial-mode degrees of freedom. Int. J. Theor. Phys..

[30] Zhang C, Sun ZW, Huang X, Long DY (2015). Three-party quantum summation without a trusted third party. Int. J. Quantum Inf..

[31] Shi RH, Mu Y, Zhong H, Cui J, Zhang S (2016). Secure multiparty quantum computation for summation and multiplication. Sci. Rep..

[32] Clementi M (2017). Classical multiparty computation using quantum resources. Phys. Rev. A.

[33] Yang HY, Ye TY (2018). Secure multi-party quantum summation based on quantum fourier transform. Quantum Inf. Process..

[34] De Vos, A. & De Baerdemacker, S. From reversible computation to quantum computation by lagrange interpolation. *arXiv e-prints* (2015).

